# Long-Term Outcomes of Nonextraction Treatment in a Patient with Severe Mandibular Crowding

**DOI:** 10.1155/2020/1376472

**Published:** 2020-08-11

**Authors:** Vincenzo Grassia, Ludovica Nucci, Paola Martina Marra, Gaetano Isola, Angelo Itro, Letizia Perillo

**Affiliations:** ^1^Multidisciplinary Department of Medical-Surgical and Dental Specialties, University of Campania Luigi Vanvitelli, Naples, Italy; ^2^Department of General Surgery and Surgical-Medical Specialties, School of Dentistry, University of Catania, Italy; ^3^Complex Operative Unit of Stomatological Surgery in Developmental Age, University of Campania Luigi Vanvitelli, Naples, Italy

## Abstract

**Objective:**

To describe a clinical case with a severe mandibular crowding treated without extraction and showing a long-term outcome.

**Methods:**

A 14-year-old boy in permanent dentition showed a class I molar and cuspid relationship, a severe deep bite of 8 mm, a constricted V-shaped upper arch with moderate crowding, and a severe crowding of about 12 mm in the lower arch. The panoramic X-ray showed an impacted upper right canine. The treatment started with the placement of a transpalatal bar and 0.022 × 0.028 in standard edgewise appliances in the upper arch and a lip bumper bonded on the second lower molars. Initial leveling of the teeth was accomplished with light Australian round wires. Finishing was then performed with rectangular wires. The phase with fixed appliances lasted 2 years and 9 months, and the patient was motivated and cooperative throughout the treatment, although with poor oral hygiene. The patient was treated without extractions.

**Results:**

The space was gained with the first and second upper molar derotations using the transpalatal bar and the gingival lip bumper in the lower arch. The upper right canine was well positioned, and the maxillary arch form was improved. The severe lower crowding was completely corrected, and a good overbite was achieved.

**Conclusion:**

A conservative, nonextraction treatment approach for this patient with class I malocclusion with severe mandibular crowding was effective, and the results have remained stable after a long-term follow-up (10 years).

## 1. Introduction

Mandibular anterior crowding represents one of the most frequent features of a malocclusion [1], and it is a critical issue due to its impact on treatment approach, prognosis, and long-term stability [2, 3].

Moreover, mandibular anterior crowding is the most common patient chief complaint.

It can be characterized by a discrepancy between the mesiodistal tooth widths of four permanent incisors and the available space in the alveolar process, although other variables such as the direction of mandibular growth, early loss of deciduous molars, incisor and molar inclinations, or perioral and oral musculature effect can occur [4].

The amount of crowding is the most important factor when deciding between extraction and nonextraction treatment [5], so the debate on which strategy can give better long-term results is still open [6]. Moreover, in the effort to identify an orthodontically ideal, long-lasting, and balanced position of the mandibular incisors, the anterior limit of the teeth [7] should always be considered.

For example, anatomic factors such as the mandibular symphysis and the labiolingual alveolar process thickness have to be evaluated [8].

Thus, in cases with narrow and high symphysis extensive orthodontic tooth movements with fixed appliances should be limited to avoid the risk of progressive bone loss of lingual and labial cortical plates [9].

Therefore, the choice of the treatment plan is greatly influenced by the morphology of the symphysis and the position of the mandibular incisors too [5–7].

Furthermore, other factors such as aesthetic features and the type of periodontium can affect the choice.

Thus, the resolution of crowding can require interproximal tooth reduction (IPR), extraction, or space gaining with increase of arch perimeter and widths and, if needed, incisor proclination [10].

One available appliance used to gain space in the mandibular arch is the lip bumper [5, 11].

The primary purpose of this appliance is to reduce dental arch crowding through an increase in arch width and length by modifying the equilibrium between the lips, cheeks, and tongue [5, 11–15, 21].

According to Buschang [14], lip bumper therapy, performed during the mixed dentition, produced useful treatment changes, with limited amounts of relapse and lasting long-term effects, which may be due to growth potential [16].

Several short-term papers confirmed this opinion along with only two long-term researches [16–18] and one clinical case [18].

However, the two systematic reviews, published later and reporting significant increase in intercanine, interpremolar, and intermolar widths, arch perimeter, and arch length concluded that the long-term effects of the lip bumper still need to be elucidated [19, 20].

Thus, it is useful for the clinician to know the long-term effects of the different treatment options offered to the patient in order to achieve the more stable result when retention is discontinued.

Many theories have been proposed although the cause of relapse is still uncertain.

According to some researches [21], final proclination and position of the mandibular incisors seem to be one of the important factors affecting stability.

On the other hand, other authors reported that the final mandibular incisor inclination and linear protrusion do not influence crowding relapse [22, 23].

In the recent years, instead, Raucci et al. showed both short- and long-term mandibular arch changes in patients treated with lip bumper followed by full fixed appliances [5]. After lip bumper treatment, they observed a significant increase in dental arch widths and perimeter along with a significant reduction in crowding and no change in arch length and incisor inclination. An average crowding of 5.39 mm was corrected with a small amount of relapse, 0.36 mm, at the follow-up lasting 6.3 years on average [5].

This clinical report shows a case with a severe mandibular crowding (12 mm) treated with a nonextraction treatment, using lip bumper followed by fixed appliance in a permanent dentition patient with a long-term outcome (10-year follow-up).

## 2. Materials and Methods

The patient was a 14-year-old boy in permanent dentition with a chief complaint of “crooked teeth.” The extraoral examination showed a narrow smile with dark corners, a very flat and retrusive profile, with retruded upper and lower lips and an increased nasolabial angle. The occlusion showed a class I molar and cuspid relationship on the left side and a class I molar on the right one where the cuspid was impacted. A severe deep bite of 8 mm and an overjet of about 2 mm were observed.

**Figure 1:**
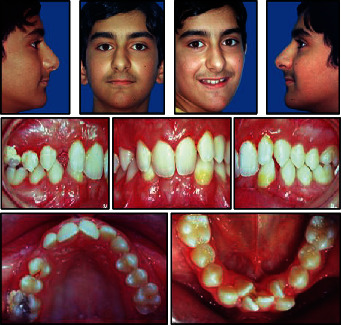
Pretreatment facial and intraoral photographs.

**Figure 2:**
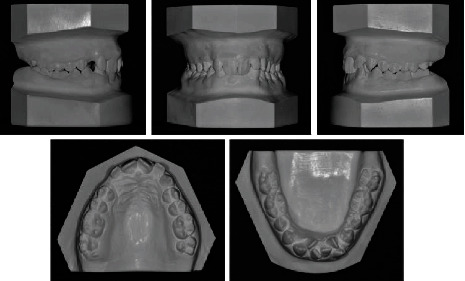
Pretreatment dental casts.

The upper arch was V-shaped with a moderate crowding, whereas a severe crowding of about 12 mm was present in the lower arch. A deep curve of Spee and lack of space for third molars were also noted (Figures 1 and 2).

The lateral cephalometric evaluation revealed a class I malocclusion (ANB, 2°) with a mesiofacial pattern (SnGoGn, 32°) and retroclined upper (1/SN, 83°) and lower incisors (IMPA, 85°) (Table 1).

Bolton analysis showed an overall ratio of 93% with lower anterior excess of 1.6 mm and an anterior ratio of 79% with a lower anterior excess of 1.2 mm.

The panoramic X-ray showed an impacted upper right canine and the presence of upper and lower third molar buds.

The impacted canine presented an angulation of 33° with respect to the midline; it was classified in the sector II according to Lindauer and sector IV according to Ericson and Kurol analysis [26, 27]. The distance between the canine cuspid and the occlusal plane was 12 mm.

All the measurements were hand traced under natural light, using 0.5 mm lead on 0.003 mm matte acetate tracing paper.

**Figure 3:**
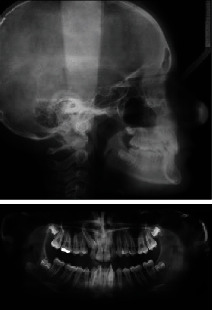
Pretreatment lateral cephalometric and panoramic X-rays.

No root resorption and bone loss were detected whereas the upper incisors showed slightly anomalous roots (Figure 3). The patient's medical and dental histories were unremarkable.

No previous orthodontic treatment was performed, and no signs or symptoms of temporomandibular joint disorder during mandibular movement were reported.

The oral hygiene was very poor with gingival inflammation in the upper and lower anterior and posterior areas. The patient's medical and dental histories were unremarkable.

The treatment objectives for this patient were to gain the space needed to guide the impacted upper right canine into the arch and to solve the crowding, to reduce the increased overbite correcting the incisor inclination, and to improve the arch forms, the smile, and the profile.

Before starting and after obtaining the informed consent from the patient's parents, we considered the following treatment options:

1. Treatment with extractions of the upper and lower first premolars
2. Treatment with extraction of a mandibular incisor, along with anterior stripping or incisor proclination, although this would create a tooth-size discrepancy of the anterior occlusion that would make correction of the overbite more difficult
3. Treatment without extractions in which in this option the space can be gained with the first and second upper molar derotations using a transpalatal bar in the upper arch and a gingival lip bumper in the lower arch positioned on the second molars. In addition, space can be gathered through the correction of the upper and lower incisor inclination with a favorable effect on smile and profile. Correct biomechanics to achieve the right torque of the lower incisors was needed
4. No treatment if not accepted by the patient's parents

The third option seemed to be the easiest and more rational choice considering the occlusal and aesthetic features and the patient's expectations.

**Figure 4:**
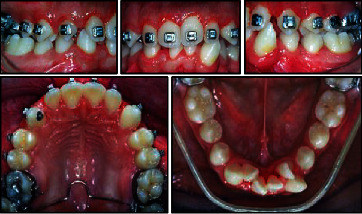
Transpalatal bar in the upper arch and lip bumper in the lower arch.

Treatment started with an interceptive first phase, including a double transpalatal bar in the maxillary arch and a lip bumper on the second lower molars (Figure 4).

After 1 year and 8 months of treatment, the upper and lower molars were derotated and an improvement of upper and lower crowding was detected; consequently, the shapes of both arches were changed.

The recovery of space in the upper arch resulted in the physiological eruption of the upper right canine.

The second phase of treatment began with the placement of 0.022 × 0.028 in standard edgewise appliances.

An intrusion arch with round Australian wire 0.016 in was used for bite opening.

The leveling of the teeth was accomplished with light Australian round wires, before 0.016 and then 0.018 in wires.

**Figure 5:**
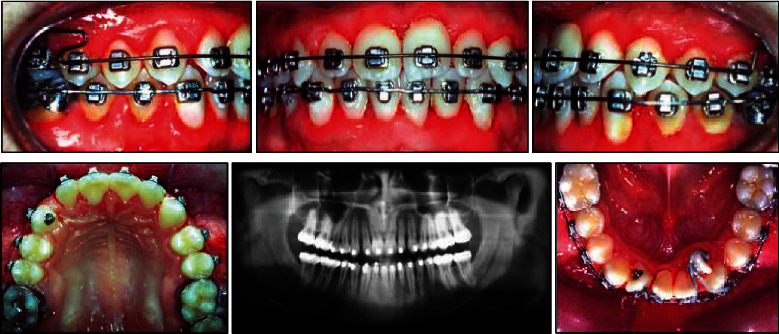
Leveling in both arches with space gaining with lateral incisor derotations in the lower arch along with panoramic X-ray before brace removal.

Open coil springs were used in the lower arch to gain space whereas lingual buttons along with elastomeric chains were applied to both lateral incisors to allow their derotation and correct placement in the arch. During the treatment, third molar bud extractions were performed. Considerable time and effort were required for the finishing procedures, including torquing, uprighting, and root paralleling (Figure 5).

After active orthodontic treatment, the brackets were removed. Maxillary and mandibular Hawley retainers were used for retention.

The fixed phase lasted 2 years and 9 months, and the patient was motivated and cooperative throughout the entire treatment.

## 3. Results

**Figure 6:**
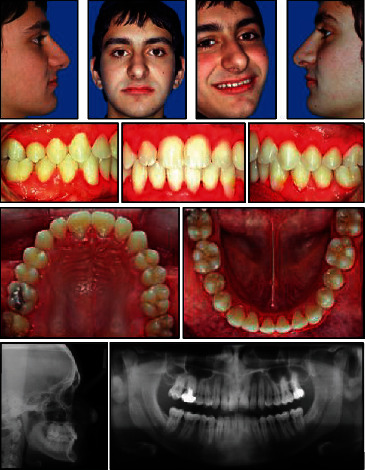
Posttreatment facial and intraoral photographs and X-rays.

**Figure 7:**
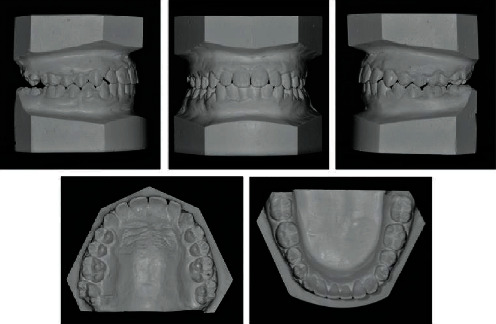
Posttreatment dental casts.

**Figure 8:**
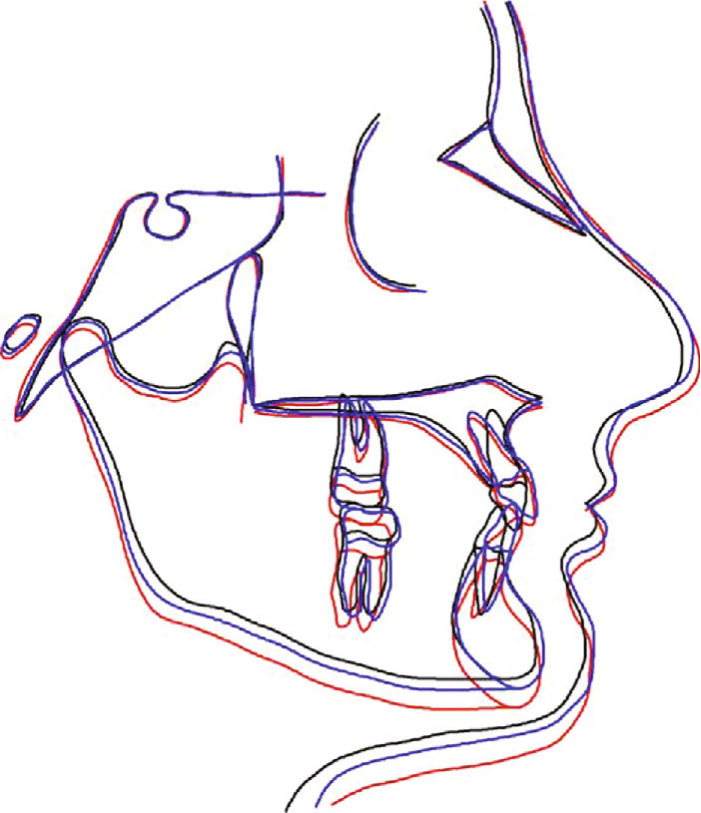
Superimposition tracings.

**Figure 9:**
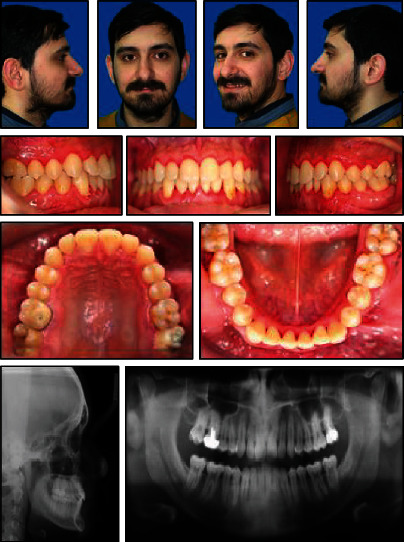
Facial and intraoral photographs, lateral and panoramic X-rays after 10-year retention.

**Figure 10:**
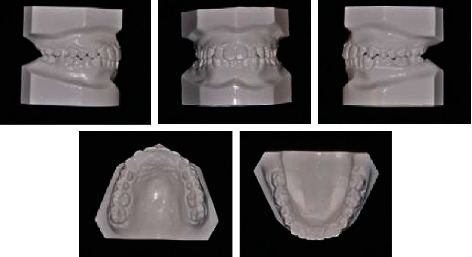
Posttreatment dental casts after 10-year retention.

The overall treatment objectives were achieved. The patient appeared with a really improved smile and profile. The occlusion showed a well-aligned dentition with class I molar and canine relationships. The patient had a consonant smile arc, interdigitation of the teeth was good, the upper right canine was well positioned, and the maxillary arch form was improved. The severe lower crowding was completely corrected, and a good overbite was achieved. The posttreatment panoramic radiograph showed nice root parallelism and the absence of the upper and lower wisdom teeth. Superimpositions revealed a proclination of upper and lower incisor inclination (1/SN, 103°; IMPA, 100°, respectively) (Figures 6–8; Table 1). The treatment lasted 4 years and 5 months and the retention period 2 years. Both the patient and his parents were pleased with the outcome. After 10 years of postretention, the occlusion was well maintained (Figures 9 and 10).

## 4. Discussion

In orthodontic diagnosis, mandibular incisor crowding is a critical and frequently a limiting factor when planning a treatment. Decisions regarding extractions in the mandible many times are greatly influenced not only by the extent of crowding but also by the relationship between basal bone and incisor position [3]. For over 6 mm of crowding, extractions seem to be the best choice whereas between 3 and 6 mm the decision is not so clear and affected by other factors such as the aesthetic and the type of periodontium.

A major goal of orthodontic treatment is to achieve a long-term stability of posttreatment occlusion [24, 25] although it is a challenge for the orthodontists; it is almost impossible to guarantee the absolute posttreatment stability, even in a case with borderline crowding [26].

For these reasons, the long-term stability after extraction or nonextraction treatment is still a controversial topic [22, 25].

According to many authors [27–32], avoiding completely the relapse is not possible in the extraction cases as well as in nonextraction cases treated in permanent dentition [32].

In particular, Cansunar and Uysal [31] pointed out that there were no significant differences among extraction and nonextraction treatments for several features such as alignment, marginal ridge height, buccolingual inclination, overjet, and interproximal contact measurements. However, nonextraction patients had both better sagittal dental relationship and root angulations [29, 33]. Moreover, in extraction treatments, the arch width reduction creates unaesthetic triangles at the corners of the mouth with negative spaces lateral to the buccal segments [32] and undesirable dental retraction; worsening facial aesthetics are possible [6]. Thus, the trend of nonextraction treatment has changed due to the new aesthetic concepts of facial soft-tissue profile in orthodontics [32–36]. The present case shows a patient with a severe mandibular crowding, treated without extractions and with a postretention long-term stability (10-year follow-up).

The severe lower crowding was completely corrected, and the improvement of overbite was obtained. The overall treatment objectives were achieved. The patient showed a better smile and profile, although the superimpositions revealed a lower incisor proclination.

A possible limitation of this treatment could be the time to gain the outcomes showed. This is due to the choice of an early treatment and the need of two phases of treatment: one in mixed dentition and one in permanent. This therapeutic option can be slowed down by the patient's dental exfoliation times. Fortunately, this strategy allows to have an early treatment, to take advantage of growth, and to prevent dental extractions.

A possible explanation for such long-term stability was that the major changes in arch dimensions were primarily achieved during the lip bumper phase, whereas the final alignment was obtained with fixed appliances [5].

So, primarily, cheek and lip muscular pressures were involved, suggesting on the one hand that gaining of space before bracket placement may help to keep the correction stable and on the other hand that a new muscular balance was achieved to allow stability of the 12 mm crowding after 10 years. The relapse of 0.5 mm in the upper and lower arch was clinically not significant.

This result can be more than satisfactory considering that the only other case report showed a mandibular crowding of 7 mm treated with lip bumper in late mixed dentition with 5 years and 3 months of follow-up including two-year retention [18].

## 5. Conclusions

An early treatment can be considered a valid choice to resolve the inclusion of a permanent canine reducing the risk of side effects and to gain space in the lower arch for the resolution of severe crowding avoiding dental extractions. The postretention stability results are remarkable because a very light relapse (0.5 mm) of the lower crowding was observed after 10 years along with an aesthetic and functional outcome.

## Figures and Tables

**Table 1:** Cephalometric measurement pre- and posttreatment.

	Pretreatment	Posttreatment	Mean SD
*Sagittal skeletal relations*			
Maxillary position S-N-A	76°	76.5°	82° ± 3.5°
Mandibular position S-N-B	74°	75.5°	80° ± 3.5°
Sagittal jaw relation A-N-Pg	2°	1°	2° ± 2.5°
*Vertical skeletal relations*			
Maxillary inclination S-N/ANS-PNS	11°	11°	8° ± 3.0°
Mandibular inclination S-N/Go-Gn	32°	32°	33° ± 2.5°
Vertical jaw relation ANS-PNS/Go-Gn	21°	21°	25° ± 6.0°
*Dentobasal relations*			
Maxillary incisor inclination 1-SN	83°	103°	110° ± 6.0°
Mandibular incisor inclination IMPA	85°	100°	94° ± 7.0°
Mandibular incisor compensation 1-A-Pg (mm)	-1.8 mm	1.0 mm	2 ± 2.0
*Dental relations*			
Overjet (mm)	2 mm	2.5 mm	3.5 ± 2.5
Overbite (mm)	8 mm	2.5 mm	2 ± 2.5
Interincisal angle 1/1	110.0°	127.0°	132° ± 6.0°
